# Retraction: Concomitant use of renin-angiotensin system inhibitors augments the efficacy of immune checkpoint inhibitors: a systematic review and meta-analysis

**DOI:** 10.3389/fphar.2025.1727252

**Published:** 2025-11-07

**Authors:** Frontiers Editorial Office

The journal retracts the 4th June 2024 article cited above.

Following concerns regarding the originality of this Frontiers in Pharmacology article, an investigation was conducted in accordance with Frontiers’ policies. It was found that the complaint was valid and that the article should be retracted due to an unacceptable level of similarity to an article published in Frontiers in Immunology on 19th April 2023 by Jinhai Shen, Hui Hou, Bowen Liang, Xiao Guo, Li Chen, Yong Yang, Yun Wang: Effect of renin-angiotensin-aldosterone system inhibitors on survival outcomes in cancer patients treated with immune checkpoint inhibitors: a systematic review and meta-analysis (Volume 14 - 2023 | https://doi.org/10.3389/fimmu.2023.1155104). Following the Committee on Publication Ethics guidelines, the article published most recently is retracted.

This retraction was approved by the Field Chief Editor of Frontiers in Pharmacology and the Chief Executive Editor of Frontiers. The authors do not agree to this retraction.

## Correction note

This Retraction Notice has been corrected to state that the authors do not agree to this retraction.

